# MsmK, an ATPase, Contributes to Utilization of Multiple Carbohydrates and Host Colonization of *Streptococcus suis*

**DOI:** 10.1371/journal.pone.0130792

**Published:** 2015-07-29

**Authors:** Mei-Fang Tan, Ting Gao, Wan-Quan Liu, Chun-Yan Zhang, Xi Yang, Jia-Wen Zhu, Mu-Ye Teng, Lu Li, Rui Zhou

**Affiliations:** 1 State Key Laboratory of Agricultural Microbiology, College of Veterinary Medicine, Huazhong Agricultural University, Wuhan, 430070, China; 2 Institute of Animal Husbandry and Veterinary, Hubei Academy of Agricultural Science, Wuhan, 430064, China; 3 Cooperative Innovation Center of Sustainable Pig Production, Wuhan, 430070, China; LSU Health Sciences Center School of Dentistry, UNITED STATES

## Abstract

Acquisition and metabolism of carbohydrates are essential for host colonization and pathogenesis of bacterial pathogens. Different bacteria can uptake different lines of carbohydrates via ABC transporters, in which ATPase subunits energize the transport though ATP hydrolysis. Some ABC transporters possess their own ATPases, while some share a common ATPase. Here we identified MsmK, an ATPase from *Streptococcus suis*, an emerging zoonotic bacterium causing dead infections in pigs and humans. Genetic and biochemistry studies revealed that the MsmK was responsible for the utilization of raffinose, melibiose, maltotetraose, glycogen and maltotriose. In infected mice, the *msmK*-deletion mutant showed significant defects of survival and colonization when compared with its parental and complementary strains. Taken together, MsmK is an ATPase that contributes to multiple carbohydrates utilization and host colonization of *S*. *suis*. This study gives new insight into our understanding of the carbohydrates utilization and its relationship to the pathogenesis of this zoonotic pathogen.

## Introduction

*Streptococcus suis* is emerging as an important zoonotic pathogen which causing deadly infections in pigs and humans. Since rapidly progressive and fatal sepsis in young pigs, infections caused by *S*. *suis* were considered a worldwide economic problem threatening the swine industry [1]. Furthermore, it has been increasingly isolated from a wide range of mammalian species including humans, and it can cause severe systemic infection with streptococcal toxic shock syndrome (STSS) [2–4]. The naso-oropharynx is one of the natural habitats for *S*. *suis* when colonizes in host, which is necessary for the pathogen to cause diseases [5,6]. In the oropharyngeal cavity of human and pigs, glucose is absorbed rapidly within 30 min after ingestion, while α-glucans can persist in large amounts [7–10].

Carbohydrates utilization is essential for bacterial growth, proliferation and pathogenesis. A large number of proteins involved in carbohydrate uptake and metabolism have been demonstrated to contribute to pathogenesis of *S*. *suis* [11–14], among the known ones is the glyceraldehyde-3-phophate-dehydrogenase (GAPDH) [15]. A recent study shows that the α-glucans catabolite of *S*. *suis* can regulate virulence gene expression in the mucosa, organs and blood [11]. It has been presented that the α-glucans utilization pathways have specific roles in pathogenic bacterial infection [16–19], while parasitic, symbiotic or fastidious bacteria appears to lack glycogen metabolism capability [20]. The transcriptome analysis of *S*. *pyogenes* has presented that α-glucans utilization occurs in deep infections of soft issues [21]. In addition, the α-glucans degradation contributes to the group A Streptococcus-host interaction [22]. Thus α-glucans breakdown and transport of the products could be essential for the *in vivo* survival and pathogenesis of *S*. *suis*.

Actually the breakdown of α-glucans by *S*. *suis* has been well studied. ApuA, a bifunctional amylopullulanase with conserved α-amylase and pullulanase, is responsible for α-glucans degradation in *S*. *suis* [12]. The α-glucans can be degraded into glucose, maltose, maltotriose and other maltodextrins, and then the products are taken up by *S*. *suis* during airway colonization. ApuA also mediates *S*. *suis* adhesion to porcine epithelium and mucus [12]. SpuA, the homolog of ApuA in *S*. *pneumoniae*, has also been demonstrated to be a virulence factor which can degrade glycogen into a ladder of maltodextrins [23]. However, carbohydrate transport remains less understood in *S*. *suis*.

Carbohydrate transport in bacteria is carried out mainly by two systems: the phosphotransferase system (PTS) and the ATP binding cassette (ABC) transporter. PTS is widely spread and relatively conserved among bacteria. Many works have been carried out to understand this system [24,25]. It is known that glucose is transported by PTS [24,26], but there is little information about transport of other carbohydrates in *S*. *suis*. The members of ABC transporter, the basic structure of which consists of two permease domains that form integral membrane channels and two ATPase subunits that energize the transport through ATP hydrolysis, are responsible for transporting a series of carbohydrates [27]. Moreover, ABC transporters from prokaryotes usually have extra solute-binding proteins (SBPs) in periplasm that are responsible for substrate capture [28].

Some ABC transporters possess their own ATPases, while some transporters share an ATPase. In *S*. *pneumoniae*, RafEFG is known to transport raffinose [29], SatABC transports sialic acid [30] and MalXCD transports maltooligosaccharides [23,31]. MsmK, a shared ATPase in *S*. *penumoniae*, energizes these carbohydrate transporters *in vivo*. Moreover, it contributes to pneumococcal colonization of *S*. *penumoniae* [32]. Another similar ABC transporter, the multiple sugar metabolism system (the Msm system), was firstly identified in *S*. *mutans*. This transporter is responsible for uptake of melibiose, raffinose, isomaltotriose, stachyose and isomaltose [33,34]. The MalXFGK transporters in *S*. *mutan*s are involved in uptake of maltotriose, maltotetraose and other maltodextrins which are zymolytic products of pullulan and glycogen [35]. The ATPases of the two ABC transporters, MsmK and MalK, can interact with either their own or the alternative transporter complex in *S*. *mutan*s [35]. Another protein MalT, an EII^mal^ PTS component, is the principal transporter of maltose and maltotriose in *S*. *mutans* [36].

In this study, we identified the gene SSUSC84_1724 which is annotated *msmK* in *S*. *suis* SC84 genome and encodes an ATPase to energize both ABC transporters MsmEFG and MalXCD. The MsmK is responsible for utilization of raffinose, melibiose, maltotetraose, glycogen and maltotriose, and contributes to *in vivo* survival and colonization of *S*. *suis*.

## Materials and Methods

### Ethics statement

All mice used in this study were purchased from the Wuhan Institute of Biological Products (Wuhan, China). The license number was SYXK(E) 2010–0029. The animal experiment protocol was approved by the Ethics Committee of Huazhong Agricultural University according to Hubei Province Laboratory Animal Management Regulations—2005. All efforts were made to minimize suffering.

### Bacterial strains, culture media and growth conditions

The bacterial strains and plasmids used in this study are listed in Table 1. *S*. *suis* strain SC-19 (streptomycin-resistant) of serotype 2 used in this study was isolated from a sick pig during the epidemic outbreak in Sichuan province of China in 2005 [37]. SC-19 and its genetically modified strains were grown in tryptic soy broth (TSB) or on tryptone soy agar (TSA) (Difco, France) supplemented with 10% fetal bovine serum (FBS) (Sijiqing, Hangzhou, China) at 37°C. For screening mutant strain Δ*msmK*, erythromyein (90 μg/ml) was added. For construction of complementary strain CΔ*msmK*, erythromyein (90 μg/ml) and spectinomycin (100 μg/ml) were added when needed. Chemically defined medium (CDM) [38] supplemented with no sugar, or 1% of glucose, maltose, maltotriose, maltotetraose, raffinose, melibiose or glycogen as indicated was used for growth assays and Western blot. Bacteria cultured with CDM were grown at 37°C.

**Table 1:** Bacterial strains and plasmids used in this study.

Strain/plasmid	Characteristics/genotype*	Source/reference
*S*. *suis*		
SC-19	*S*. *suis* serotype 2, the wild-type (Strep^r^)	(37)
Δ*msmK*	SC-19 *msmK*::*erm* (Strep^r^ Erm^r^)	This study
CΔ*msmK*	SC-19 *msmK*::*erm/msmK* ^+^ (Strep^r^ Erm^r^ Spc^r^)	This study
*E*. *coli*		
DH5α	F^-^ *endA1 glnV44 thi-1 recA1 relA1 gyrA96 deoR nupG* Φ80d*lacZ*ΔM15 Δ(*lacZYA*-*argF*)U169, *hsdR17*(r_K_^-^m_K_^+^), λ–	Trans
BL21(DE3)	F^-^ *ompT hsdS*_*B*_(r_B_^-^m_B_^-^) *gal dcm* (DE3)	Trans
Plasmids		
pET-28a	Expression vector (Kan^r^)	Novagen
pMsmK	pET-28a containing *msmK*, cloned from SC-19 genome	This study
pAT18	A plasmid carrying an erythromycin resistance rRNA methylase (*erm*) gene expression cassette	(40)
pSET4s	Temperature-sensitive *E*. *coli—S*. *suis* shuttle vector (Spc^r^)	(39)
pSET4s-M	Derived from pSET4s for deleting *msmK* in SC-19	This study
pSET2	*E*. *coli—S*. *suis* shuttle vector (Spc^r^)	(42)
pSET2-CM	Derived from pSET2 for functional complementation of Δ*msmK* (Spc^r^)	This study

* Strep^r^, streptomycin resistant; Erm^r^, erythromycin resistant; Spc^r^, spectinomycin resistant; Kan^r^, kanamycin resistant.

*E*. *coli* DH5α (Trans, China) was used as a host strain for cloning, and *E*. *coli* BL21 (DE3) (Trans) was used as a host strain to express His-tag fusion protein MsmK. Both were cultured in Luria-Bertani (LB) broth (Difco) or plated on LB agar plates at 37°C or 18°C.When appropriate, kanamycin (25 μg/ml) was added for bacterial selection.

Unless otherwise specified, all the antibiotics, chemicals and substrates were purchased from Biosharp (Hefei, China).

### Mutagenesis and genetic complementation

Thermosensitive suicide vector pSET4s was used for gene replacement in *S*. *suis* through homologous recombination [39]. All the primers were designed according to the genome sequence of *S*. *suis* SC84 (a human-origin strain isolated in the 2005 outbreak of Sichuan China, with similar background as strain SC-19 used in this study, GenBank accession number NC_012924.1) and are listed in Table 2. Two pairs of specific primers (Mup-F/Mup-R and Mdown-F/Mdown-R) were used for cloning the *msmK* up- and downstream homologous regions, and then cloned into pSET4s. The *erm* gene expression cassette was amplified from pAT18 [40] by using primers Erm-F/Erm-R and inserted between the up- and downstream homologous arms in the recombinant pSET4s to obtain pSET4S-M. To acquire isogenic mutant Δ*msmK*, SC-19 was transformed by electroporation of pSET4S-M as described previously [41]. The Δ*msmK* strain was identified on TSA plates for its erythromyein resistance and sensitivity to spectinomycin.

**Table 2:** Oligonucleotide primers used in this study.

Primers	Primers sequence (5’—3’)*	Amplification for
Mup-F	GCAAGCTTATGTCGGTTACTTCTATAAGAT	Upstream border of *msmK*
Mup-R	GGAGTCGACGTTCAATCCTCGCTTTCTTT	
Mdown-F	GCGGATCCCCTTTCTAAAATCGTTCATAAA	Downstream border of *msmK*
Mdown-R	CTAGAGCTCACCGTTTCAACTGGGAGG	
Erm-F	GCGTCGACCTTAGAAGCAAACTTAAGAGT	Erm^R^ expression cassette
Erm-R	TACGTCGACATCGATACAAATTCCCCGTAG	
CM-F	GTGCATGCTAGGAGCTAATGATGAGAAAAG	*msmK* coding sequence and its promoter
CM-R	GTGCATGCTTAGACAATCGCCTTGCTTGTT	
M-F	CGGGATCC TTGAACATGGTTCAATTGAATT	*msmK* coding sequence
M-R	CGCTCGAGTTAGACAATC GCCTTGCTTG	
16SrRNA-F	GTAGTCCACGCCGTAAACG	Real-time PCR for 16S rRNA
16SrRNA-R	TAAACCACATGCTCCACCGC	
MsmE-F	CTACCTATGCAGAATTTGTGG	Real-time PCR for *msmE*
MsmE-R	CCTTTGCACTACGAATCAGAAT	
MalX-F	CTTGGTTCTGAAGGTCAACTAT	Real-time PCR for *malX*
MalX-R	CAAGTTCTGCAAATGTCTTAGG	
MalT-F	GGTCTTGCCTTCATCCTTATT	Real-time PCR for *malT*
MalT-R	GAAGTAGCCTGAAACTGGAAT	
MsmK-F	TCTTAGAACGTAAACCAGCTG	Real-time PCR for *msmK*
MsmK-R	TAGCAATCTCTGTACGCATGG	

* Underlined nucleotides denote enzyme restriction sites.

To obtain a complementary strain, the promoter and coding sequence of *msmK* gene was amplified by PCR using specific primers CM-F/CM-R and then cloned into pSET2 [42] to obtain the recombinant plasmid pSET2-CM, which was electroporated into Δ*msmK*. The complementary strain CΔ*msmK* was screened and confirmed on TSA plates for erythromyein and spectinomycin resistance.

### Preparation of recombinant MsmK

The *msmK* coding sequence was amplified from SC-19 genomic DNA with primers M-F/M-R, and cloned into the prokaryotic expression vector pET-28a. The resultant plasmid pMsmK was transformed into *E*. *coli* BL21 (DE3) for expression by induction with 1 mM of isopropyl-β-D-thiogalactopyranoside (IPTG; Sigma, USA) overnight at 18°C. His-tagged MsmK protein (His-MsmK) was purified by using Ni-NTA columns (GE Healthcare, USA) and desalted in phosphate-buffered saline (PBS) buffer with centrifugal filters (Millipore, USA). Quality and quantity of the purified protein was tested by SDS-PAGE and BCA method (Micro BCA protein assay kit; Cwbiotech, China), respectively. Finally, the purified protein was stored at -80°C.

### ATPase assay

ATP hydrolysis by MsmK was assayed as described previously [43]. Calculated MsmK was preincubated for 10 min at 30°C in buffer P (50 mM PIPES/NaOH, pH6.8, 200 mM KCl, 10 mM MgCl_2_, 1 mM β-mercaptoethanol). A 25-μl reaction system was started by addition of [α-^32^P]-ATP (3 Ci/mol; PerkinElmer, UK) to a final concentration of 1 mM. After incubated at 30°C for 1 h, 2.5 μl samples were taken and transferred onto TLC PEI Cellulosose F plastic sheets (Merck, Germany). The sheets were developed in a solvent buffer containing 1 M LiCl and 0.5 M formic acid in H_2_O and exposed to a phosphor screen (GE Healthcare). After scanning the screen in a phosphor imaging analysis system (Fujifilm, Japan), the amount of [α-^32^P]-ADP was quantified using ImageJ2x and used to calculate the overall amount of ADP generated in the reactions. Bovine serum albumin (BSA) was used as negative control. Each assay was performed in triplicate.

### Fermentation assays

The API-20 STREP kit and the API Staph Strip kit (Biomérieux, France) were used in this study following the manufacturers’ recommendations and the methods described previously [44–46]. The SC-19, Δ*msmK*, CΔ*msmK* bacterial suspensions were adjusted to a turbidity between MacFarland Tubes 5 and 6. The two galleries include the following carbohydrate tests: D-glucose, D-fructose, D-mannose, maltose, lactose, D-trehalose, D-mannitol, xylitol, sucrose, D-melibiose, D-raffinose, D-xylose, D-saccharose, alpha-methylglucoside, N-acetylglucosamine, D-ribose, L-arabinose, D-sorbitol, inulin, starch and glycogen. The galleries were inoculated and incubated at 37°C for 24 h as recommended. The results were affirmed according to the color presented: red means positive while yellow means negative. Each assay was performed in triplicate.

### Growth assays

Growth assays were processed with integrating methods described previously [30,32,47]. *S*. *suis* strains were grown in TSB to the mid-exponential phase (an OD_600_ of 0.6). Cells were collected and washed in PBS buffer thrice by centrifugation at 6,000 × *g* for 10 min. Each sample was resuspended in CDM with no/disparate sugar to an OD_600_ of 0.1~0.2 and then incubated at 37°C for 8 h. OD_600_ was measured hourly with a Biophotometer (Eppendorf, Germany). CDM containing 1% glucose was used as positive control in all experiments to confirm the viability of strains. CDM with no sugar was served as negative control. Data collected were calculated and drafted with Graphpad Prism 6 software (Graphpad, USA). All experiments were conducted in duplicate and repeated at least three times.

### RNA extraction and quantitative RT-PCR (qRT-PCR)

For RNA extraction, SC-19 was grown to early stationary phase in CDM supplemented with 1% glucose, 1% raffinose, 1% melibiose, 1% glycogen, 1% maltotrise or 1% maltotetraose. One-ml culture was centrifuged and collected by centrifugation at 12,000 × *g* and 4°C. RNA was isolated with SV Total RNA Isolation System (Promega, USA) according to the manufacturer’s instructions. The quality and concentration of the total RNA were assessed with the Biophotometer (Eppendorf) and by the A_260_/A_280_ ratio. cDNA was synthesized using HiScript Q Select RT SuperMix (Vazyme, China) according to the manufacturer’s instructions. Parallel samples were processed without the addition of reverse transcriptase as a negative control.

For qRT-PCR, primers were designed using Oligo Program version 6 (MedProbe, Norway) and are listed in Table 2. The SYBR green master mix (Vazyme) was used according to the manufacturer’s instructions. Reactions were performed on an ABI 7500 system with the following parameters: 95°C for 10 min, 40 cycles of 95°C 30 s, 57°C30 s and 72°C 35 s, followed by a melting curve analysis. 16S rRNA was used as an internal control. Primers targeting the coding sequences of *msmE*, *malX*, *malT* and *msmK* were used for analysis. The relative level of expression was calculated using the 2^-ΔΔCt^ method as described previously [48], and the data were analyzed with Graphpad Prism 6 software. The experiment was performed in twice and three replicates of all samples. Non-template controls were included for each gene in each run.

### Western blot

Enolase was used as the internal reference for its stable expression levels cultured *in vitro* [11]. Western blot was performed with mouse anti-enolase serum [49] and mouse anti-MsmK serum which was generated according to the method described in our previous report [50]. The overnight cultures of SC-19, Δ*msmK* and CΔ*msmK* cultured in TSB or SC-19 cultured in CDM were collected by centrifugation at 12,000 × *g* for 10 min and resuspended in bacterial lysis buffer (50 mM Tris/HCl, pH 8.5, 100 mM NaCl, 2 mM ethylene diamine tetraacetie acid, 100 μg/ml lysozyme, 1 mM phenylmethanesulfonyl fluoride, 0.5% Triton X-100). Bacterial cells were lysed with a French pressure cell press and supernatant was preserved for Western blot analysis by centrifugation at 12,000 × *g* and 4°C for 30 min. Quantified bacterial lysate was separated on SDS-PAGE and then electrotransferred to PVDF membrane (Invitrogen, USA). The membrane was blocked in 5% skim milk in TBST buffer (20 mM Tris/HCl, pH8.0, 150 mM NaCl, 0.05% Tween-20) at 4°C overnight. After washed thrice with TBST buffer, the membrane was probed at 37°C for 2 h with 1:1000 diluted mouse anti-MsmK serum or mouse anti-Enolase serum. After three times washing the membrane was incubated with goat anti-mouse IgG (H+L)-HRP (1:5000) (Southern Biotech, USA) at 37°C for 1 h. Detection was carried out by using Western ECL Substrate Kit (Bio-Rad, USA) and ECL Plus Western Blotting Detection System (DNR, Israel). Graphics were quantified with ImageJ2x to calculate the MsmK amount contained in whole bacterial proteome. All experiments were done in triplicate.

### Murine colonization

Murine colonization assay was performed as described previously [32,47]. Groups of 25 female specific-pathogen-free (SPF) Kun-Ming mice (4 to 6 weeks old) were inoculated by intraperitoneal injection with 3 × 10^7^ mid-log-phase cells of a 1:1 mixture of SC-19 and Δ*msmK*. Five control mice were inoculated with saline. 6 h, 12 h, 1 day, 3 days and 5 days after inoculation, mice were sacrificed by carbon dioxide asphyxiation. The brains were obtained and 5 mice were sacrificed at each time point. Brain samples were placed in 1 ml PBS and homogenized after weighed. Then serial dilutions were plated onto TSA plates. TSA with 20 μg/ml streptomycin was prepared for the wild type SC-19, while TSA with streptomycin and erythromycin was selective for the mutant strain Δ*msmK*. Colonies were counted and expressed as CFU/g tissue. Date collected were drafted and analyzed by Graphpad Prism 6 software.

To prove whether the defected colonization of the mutant was not caused by weakened proliferation during the very early colonization stage, an *in vitro* competition assay was carried out as described previously [32]. SC-19 and Δ*msmK* were grown in TSB with 10% FBS to an OD_600_ of 0.6. Then they were 1:1 mixed (2 x 10^7^ CFU/ml, respectively) and incubated in fresh TSB containing 10% FBS at 37°C for 4 h. Bacteria were enumerated by serial dilution and plated onto TSA plates with/without erythromycin. Colonies were counted and the ratio of the CFU of mutant to parental strain was caculated.

## Results

### Genetic organization of MsmEFG and MalXCD transporters in *S*. *suis* genome

**Fig 1:**
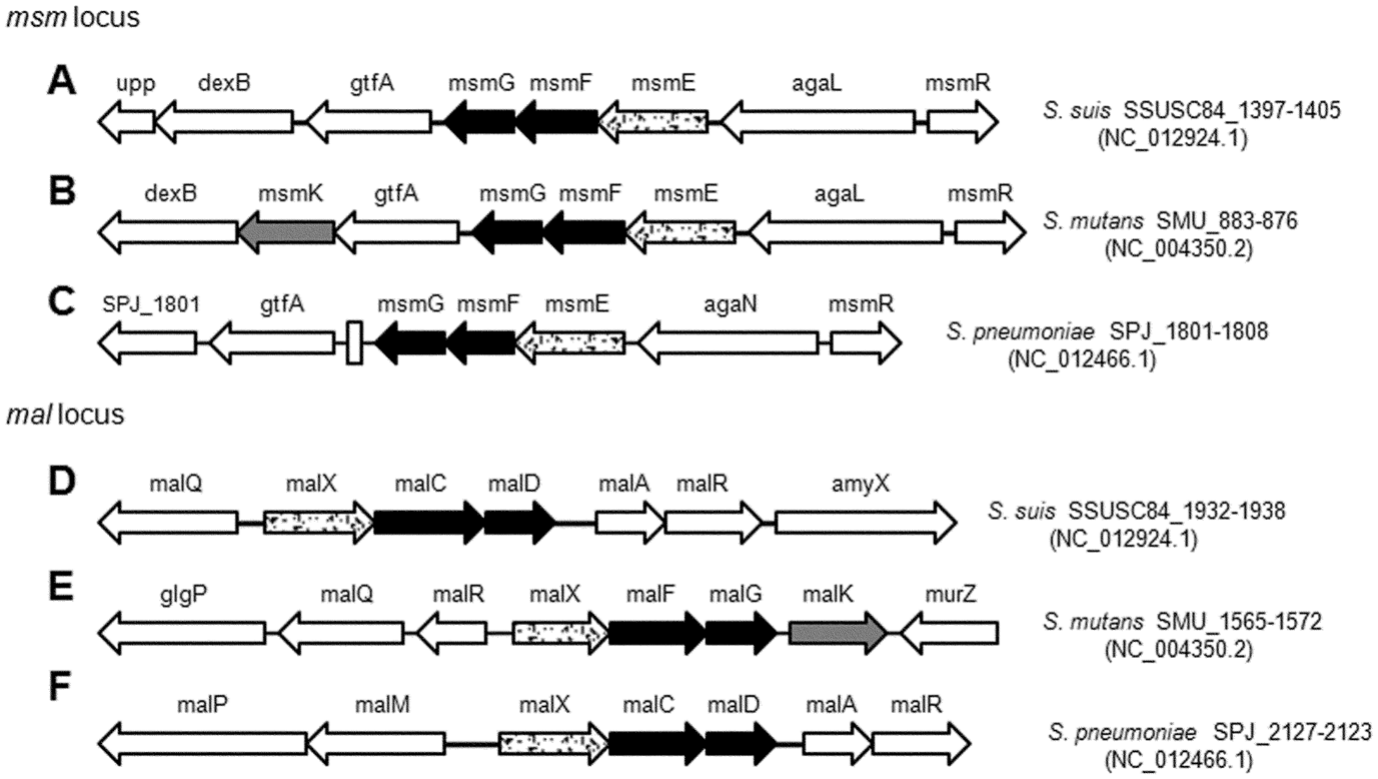
Schematic presentation of the *msm* and *mal* loci encoding relevant carbohydrate ABC transporters in selected streptococci. The genes within the loci encoding components of the *S*. *suis* MsmEFG (A), *S*. *mutans* MsmEFGK (B), *S*. *pneuminiae* MsmEFG (C), *S*. *suis* MalXCD (D), *S*. *mutans* MalXFGK (E) and *S*. *pneumoniae* MalXCD (F) are represented. Arrows indicate the direction of transcription. Gray arrows, genes encoding the ATPase of ABC transporters; spotted arrows, genes encoding solute binding proteins; black arrows, genes encoding permeases of ABC transporters; blank arrows, other genes adjacent.

The CUT1, CUT2 and peptide/opine/nickel uptake transporter (PepT) subfamily are three subfamilies classified as ABC transporters [51]. The genome of SC84 includes two regions encoding the CUT1 subfamily of ABC transporters: MsmEFG and MalXCD (Fig 1A and 1D), both of which consist of a solute binding protein (MsmE and MalX) and two membrane proteins (MsmF/MsmG and MalC/MalD).

The *msm* gene cluster and adjacent genes in *S*. *suis* has similar alignment with *S*. *mutans* except for lack of *msmK* in the locus (Fig 1A and 1B). Amino acid sequence alignment indicates high similarity between Msm proteins in *S*. *suis* and *S*. *mutans*: MsmE (60%), MsmF (54%) and MsmG (63%). Since the MsmEFG transporter in *S*. *mutans* is involved in the uptake of distinct carbohydrates including raffinose and melibiose [35], we speculated that MsmEFG transporter may also play a role in transporting raffinose and melibiose in *S*. *suis*. As predicted, relative to bacteria grown in glucose, expression of *msmE* significantly increased in the presence of raffinose and melibiose (Table 3), indicated that the MsmEFG transporter contributes to transport of raffinose and melibiose.

**Table 3:** Fold changes in transcription of transporter genes compared to glucose-grown bacteria by qRT-PCR.

Gene	Fold change (± SD)*				
	Raffinose	Melibiose	Glycogen	Maltotrise	Maltotetraose
*msmE*	**121.10±41.28**	**86.82±28.78**	4.89±2.76	3.43±0.07	-0.73±2.78
*malX*	8.17±2.22	2.30±1.53	**13.09±3.86**	**15.56±2.08**	**13.93±1.45**
*malT*	1.71±0.45	1.77±2.41	**95.01±20.35**	**89.88±9.68**	**52.71±17.64**

* Bolded text indicates the substrate binding protein expression in the corresponding medium.

The *mal* gene cluster in *S*. *suis* is highly homologous to that in *S*. *pneumoniae* and *S*. *mutans* (Fig 1D-1F). The MalXFG transporter in *S*. *mutans* and MalXCD transporter in *S*. *penumoniae* have been demonstrated to be responsible for taking up maltodextrins [23,32,35]. Through amino acid sequences alignment, the identity of Mal proteins in *S*. *suis* and *S*. *pneumoniae* are 68% (MalX), 84% (MalC) and 85% (MalD), respectively. Relatively lower homology of Mal proteins was discovered between *S*. *suis* and *S*. *mutans*. Through qRT-PCR, *malX* expression increased at least 10-fold when SC-19 was grown in glycogen or maltodextrins compared with glucose, which demonstrated that MalXCD transporter was responsible for uptake of maltodextrins in *S*. *suis*.

**Fig 2:**
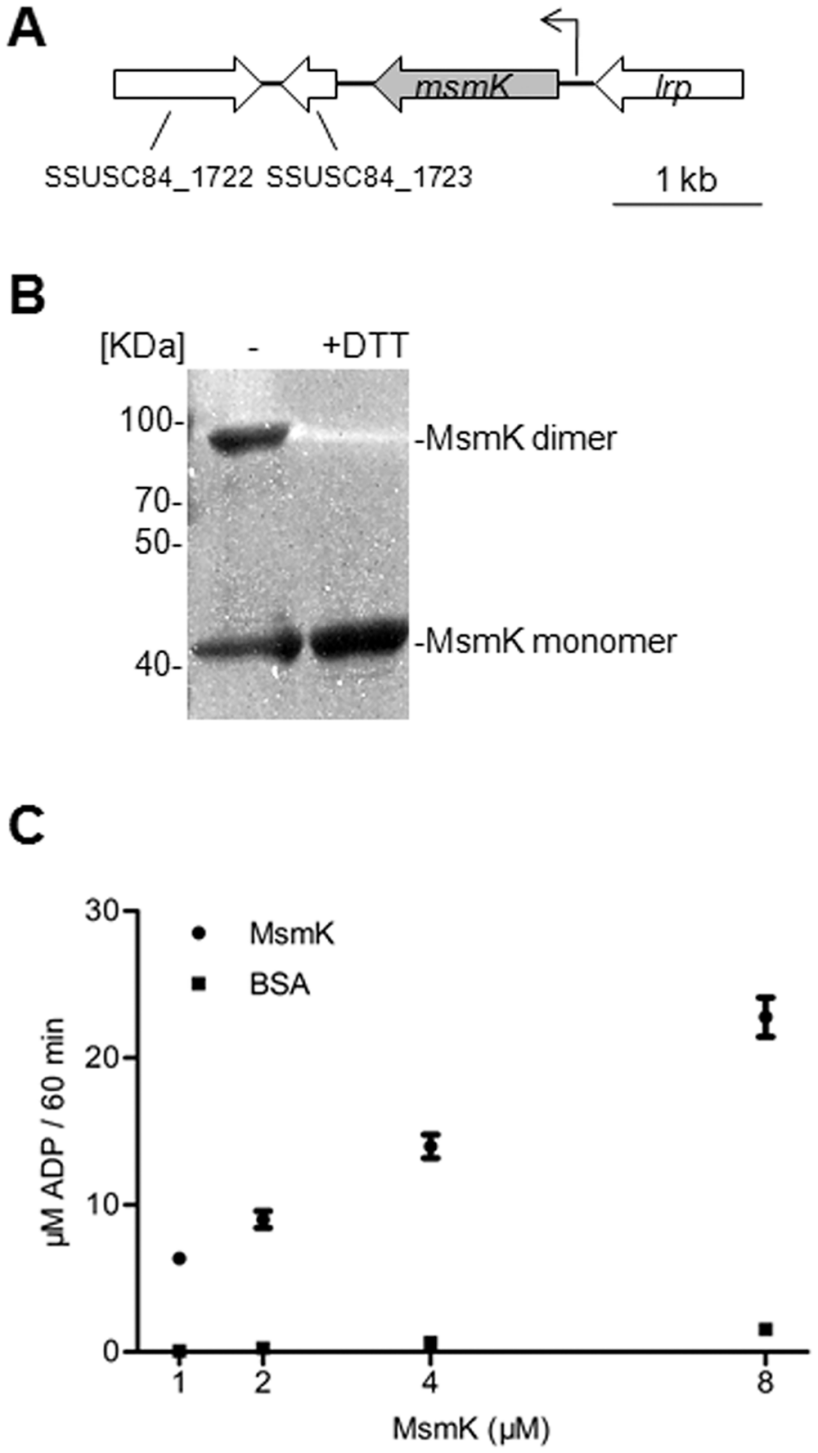
MsmK is an ATPase. (A) Genetic map of the loci encoding predicated carbohydrate ATPase, MsmK, in SC84. Large arrows represent open reading frames and their direction of transcription. Small arrow indicates the promoter of the gene *msmK* and its transcription direction. (B) SDS-PAGE of purified recombinant protein MsmK. Before electrophoresis, the proteins were exposed to no DTT (left lane,-) or 2 mM DTT (right lane, +DTT). Purified MsmK was formed by a certain amount of monomers (42 KDa) and dimmers (~80 KDa). (C) ATPase activity of MsmK. Protein was incubated with 1 mM ATP for 60 min, and the generation of ADP was quantified. The mixture with BSA protein was used as a negative control. Data represent the average ± SD of at least three independent repeats.

Like most operons encoding CUT1 transporters in Streptococci species (such as *S*. *pneumoniae* in Fig 1C and 1F), there is no ATPase gene in the operon. Given that ATPase is an essential component of an ABC transporter to energize the substrate transport, an ATPase gene must exist in other locus or loci in the genome of these Streptococci species. It is known that in *S*. *mutans*, MsmK and MalK function as ATPase for the MsmEFG and MalXFG transporters, respectively [35], while MsmK energizes multiple carbohydrates transporters in *S*. *pneumoniae* including the MalXCD transporter [32]. To identify the ATPase which energizes the MsmEFG and MalXCD transporters in *S*. *suis*, we screened *S*. *suis* genome sequences available in GenBank and found that the protein encoded by SSUSC84_1724 in the genome of *S*. *suis* strain SC84 showed the highest similarity to the MsmK of *S*. *pneumoniae* (79.5%) and *S*. *mutans* (78.5%) (Fig 2A) [30,32,33,35]. It contains the typical ATPase domains as MsmK: Walker A, Q-loop, ABC transporter signature motif, Walker B, D-loop and H-loop (S1 Fig) [27,52]. Taken together, we speculate that the SSUSC84_1724 gene encodes an ATPase for the MsmEFG and MalXCD transporters of *S*. *suis*, and rename it as MsmK.

### ATPase activity of *S*. *suis* MsmK

To validate that SSUSC84_1724 encodes an ATPase, its homologous gene was cloned from SC-19 and expressed in *E*. *coli*. The recombinant protein could form dimer spontaneously *in vitro* which could be degraded into monomer by DTT (Fig 2B). ATPase assays showed that the recombinant protein could hydrolyze ATP to ADP (Fig 2C), indicating that the MsmK is indeed an ATPase.

### MsmK is required for *S*. *suis* growth using raffinose, melibiose, glycogen or malitrotetraose as sole carbon source

To further investigate the function of MsmK in *S*. *suis*, isogenic mutant strain Δ*msmK* and complementary strain CΔ*msmK* were constructed, and confirmed by Western blot (S2 Fig). Fermentation assays were carried out for 21 kinds of carbohydrates contained in the API-20 STREP kit and API Staph Strip kit. According to the color changes, Δ*msmK* failed to use raffinose, melibiose and glycogen as carbon source, while SC-19 and CΔ*msmK* were able to ferment these three kinds of carbohydrates (Table 4).

**Table 4:** MsmK affects fermentation of different carbohydrates in *S*. *suis*.

Carbohydrate	SC-19	Δ*msmK*	CΔ*msmK*
D-glucose^a^	+^d^	+	+
D-fructose	+	+	+
D-mannose	+	+	+
Maltose	+	+	+
Lactose	+	+	+
D-trehalose	+	+	+
D-mannitol	-^e^	-	-
Xylitol	-	-	-
Sucrose	+	+	+
D-xylose	-	-	-
D-saccharose	+	+	+
Methyl-αD-glucopanpside	-	-	-
N-acetyl-glucosamine	+	+	+
D-ribose	-	-	-
L- arabinose	-	-	-
D-Sorbitol	-	-	-
Inulin	-	-	-
Starch	+	+	+
Glycogen ^b^	+	-	+
D-raffinose ^b^	+	-	+
D-melibiose ^b^	+	-	+
0 ^c^	-	-	-

^a^ glucose used as the positive control

^b^ the mutant cannot utilize the carbohydrate any more compared with the parental strain.

^c^ no substrate added in reaction system, used as the negative control.

^d^ fermentation result is positive, the strain could utilize the carbohydrate for acid production.

^e^ fermentation result is negative, the strain couldn’t utilize the carbohydrate for acid production.

**Fig 3:**
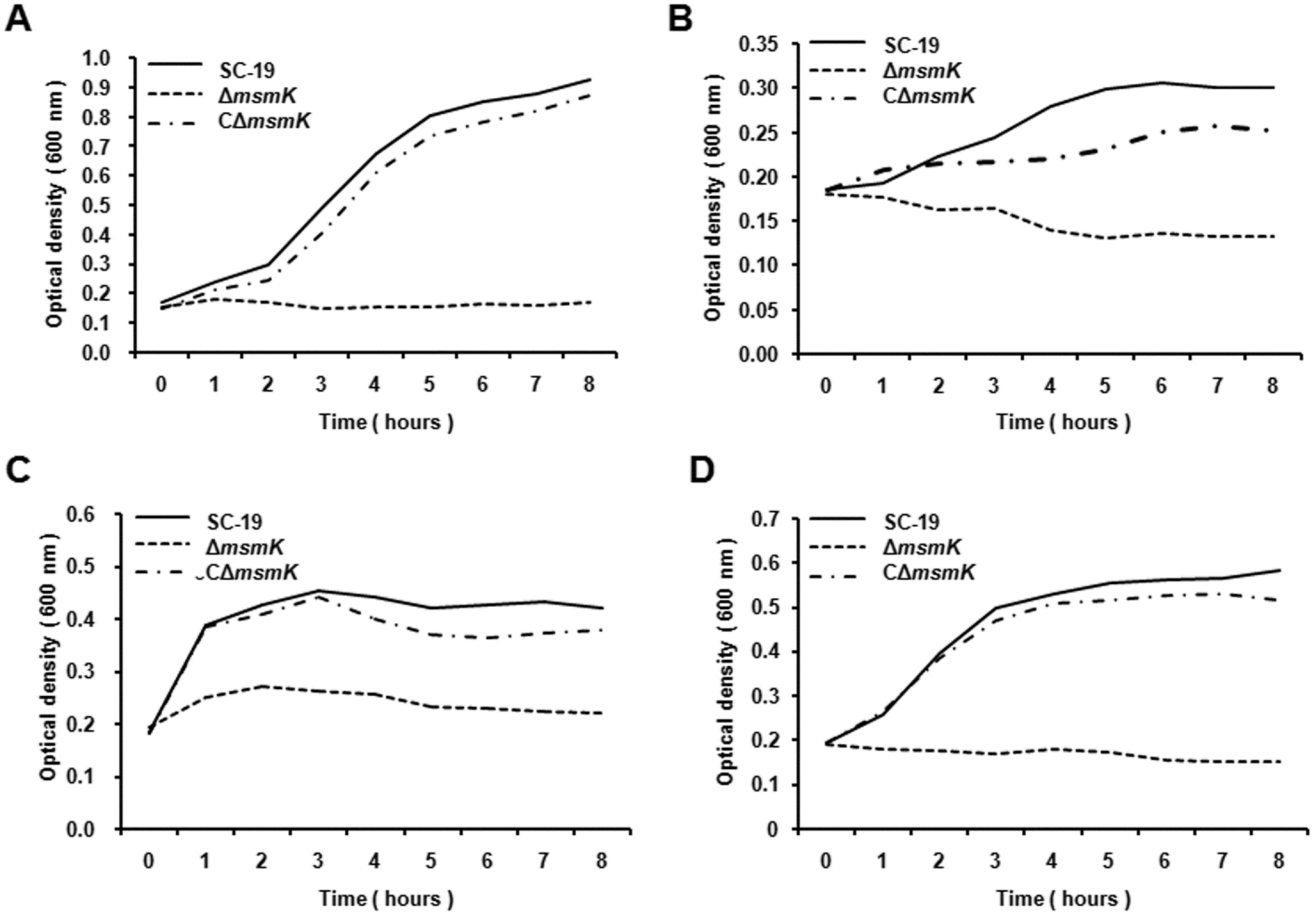
MsmK is required for growth in raffinose, melibiose, glycogen and maltotetraose. The SC-19, Δ*msmK* and CΔ*msmK* strains were grown in CDM containing 1% raffinose (A), 1% melibiose (B), 1% glycogen (C) or 1% maltotetraose (D) as sole carbon source. Growth was measured by the optical density at 600 nm. Data are presented as means from at least three independent experiments.

Additionally, deletion of *msmK* resulted in dramatically growth inhibition compared to the wild type strain when cultured in CDM containing raffinose, melibiose or glycogen (Fig 3A–3C) as sole carbon source. Since MsmK energizes the MalXCD transporters to transport maltodextrins in *S*. *pneumoniae* [23,32], Δ*msmK* was also tested for growth in CDM with maltotetraose as sole carbohydrate. The results showed that the mutant was unable to grow in this medium (Fig 3D). CΔ*msmK* completely or partially restored the growth defect in all growth assays. These results demonstrate that MsmK is responsible for utilization of raffinose, melibiose, glycogen and malitrotetraose in *S*. *suis*, which also imply that MsmK energizes the MsmEFG and MalXCD transporters in *S*. *suis*.

### Growth of Δ*msmK* is suppressed by maltotriose as sole carbon source

**Fig 4:**
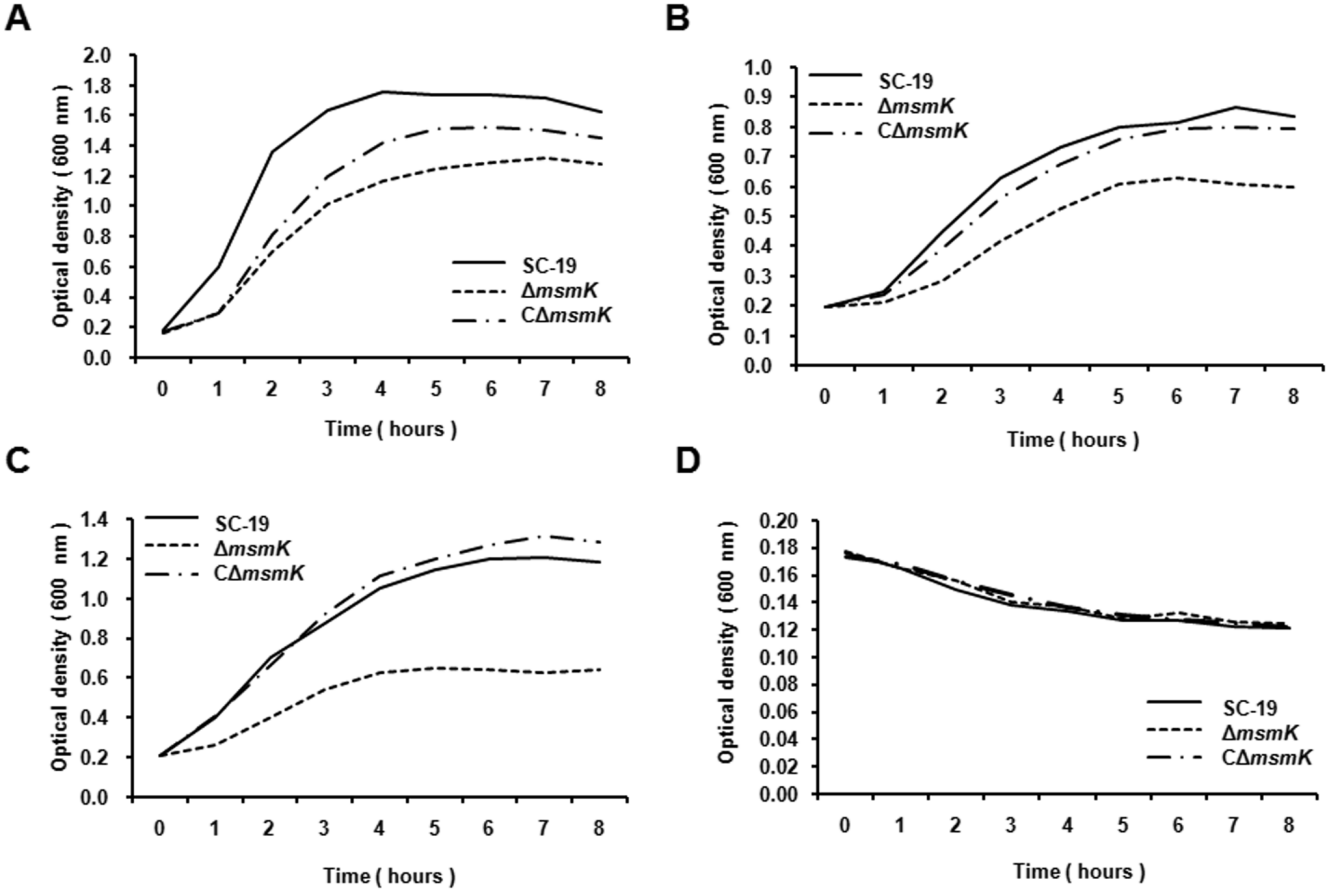
Deletion of *msmK* impacts bacterial growth on maltotriose. The SC-19, Δ*msm*K and CΔ*msmK* strains were grown on the CDM containing 1% glucose (A), 1% maltose (B), 1% maltotriose (C) or no sugar (D) as sole carbon source. CDM supplemented with glucose/no sugar served as a positive/negative control respectively in growth assays. Growth was measured by the optical density at 600 nm. Data are means from at least three independent experiments.

To detect if MsmK contributes to the utilization of other carbohydrates, *S*. *suis* strains were cultured in CDM supplemented with glucose, maltose or maltotriose as sole carbon resource (Fig 4A–4C). CDM supplemented with no sugar served as negative control in growth assays (Fig 4D). Interestingly, the mutant displayed growth defect in the CDM containing glucose compared with the parental strain according to the OD values (Fig 4A), but there was no significant difference between the two strains when measuring the numbers of living bacterium at stable stage (S3 Fig). The same phenomenon was observed on the mutant when cultured in maltose (Fig 4B). Unlikely in glucose and maltose, the growth of Δ*msmK* was largely inhibited when using maltotriose as sole carbon source (Fig 4C).

### MsmK expression is induced by multiple carbohydrates

**Fig 5:**
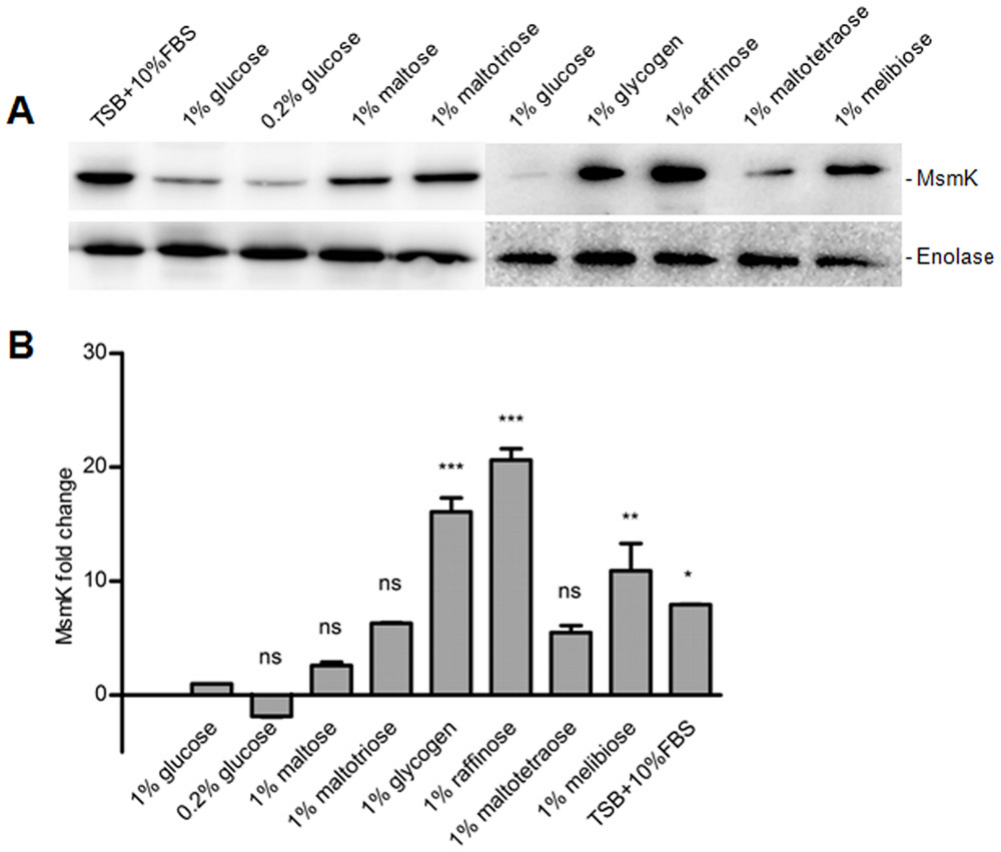
Western blot analysis of MsmK expression in SC-19 cultured in different carbohydrates. (A) Strain SC-19 was cultured in CDM supplemented with 1% (g/v) diverse carbohydrates to log phase. The supernatant of cell lysate was disposed for immunoblot analysis with MsmK or Enolase polyclonal antibodies. Enolase was used as an internal reference. (B) The blots were scanned, and the resultant graphics were quantified with ImageJ2x. Fold change is represented as a comparison to cells grown in glucose, and the expression levels of Enolase as internal references. Data are shown as means ± SEM from at least three independent experiments. Statistical significance was tested by a Two-way ANOVA test (ns, *P* > 0.05; _*_, *P* ≤ 0.05; _**_, *P* ≤ 0.01; _***_, *P* ≤ 0.001).

Since MsmK is suspected to energize the MsmEFG and MalXCD transporters in *S*. *suis* for multiple carbohydrates transport, it was supposed that the levels of MsmK expressed in CDM with diverse carbohydrates aforementioned would be induced in some degree. To obtain the relative expression levels of MsmK, SC-19 was cultured in CDM supplemented with different carbohydrates as sole carbon source. MsmK expression was evaluated by Western blot (Fig 5A). The experimental images (Fig 5A) were quantified by using ImageJ2x software. The amount of MsmK expressed in SC-19 cultured in CDM with glucose was defined as one fold. As expected, expression of MsmK increased on different levels (Fig 5B). According to the result, expression of MsmK in 1% melibiose, glycogen and raffinose were significantly increased (*p* ≤ 0.01). In contrast, levels of MsmK in 1% maltotriose and maltotraose had fewer changes, but still higher than that in 1% glucose or maltose. MsmK expression induced by these carbohydrates further supports the assumption that MsmK contributes to multiple carbohydrates in *S*. *suis*.

### MsmK contributes to *in vivo* survival and colonization of *S*. *suis*

**Fig 6:**
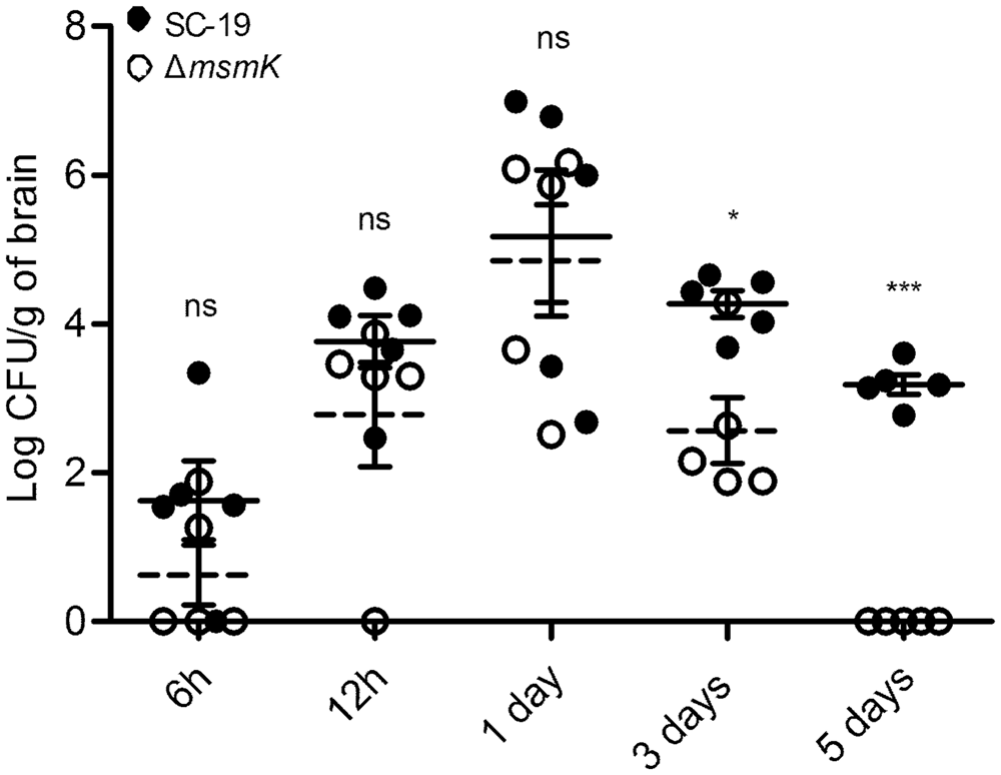
MsmK contributes to streptococcal survival in brain. Mice were received intraperitoneal injection with 3 × 10^7^ CFU of a 1: 1 mix of SC-19 and Δ*msmK* strains. At each defined time point, brains of 5 mice were collected and the viable bacteria were counted. The SC-19 and Δ*msmK* strains were distinguished by erythromycin added in TSA plates. Solid lines, the means of data of strain SC-19; dotted lines, the means of data of strain Δ*msmK*. The means and standard errors of the means are depicted. Statistical significance was tested by a two-tailed Student *t* test (_*_, *P* ≤ 0.05; _***_, *P* ≤ 0.001).

To study the functions of MsmK in pathogenesis, Δ*msmK* and its parental strain SC-19 were equally mixed and inoculated into mice. For meningitis is the most striking feature caused by *S*. *suis* and often the basis of a presumptive diagnosis [18], the bacterial loads of two strains in brain were measured at different time points post infection. Data showed that the numbers of the mutant were significantly decreased than that of the wild type strain in brain from the third day (Fig 6). These results indicate that MsmK contributes to *in vivo* survival and colonization of *S*. *suis* in mice.

## Discussion

**Fig 7:**
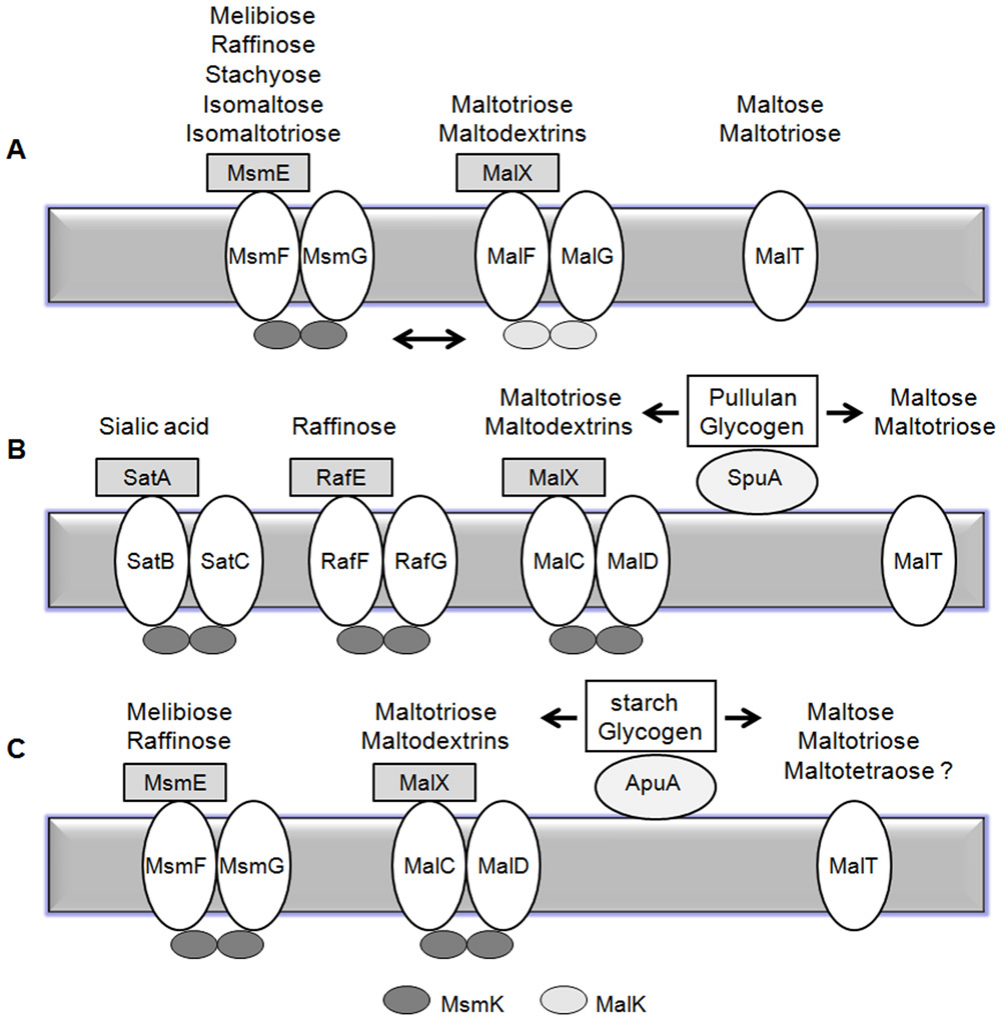
Schematic summary of carbohydrates utilization by ABC transporters in *S*. *mutans* (A), *S*. *pneumoniae* (B) and *S*. *suis* (C). Above each ABC complexes is a list of known or putative carbohydrates transported by each ABC transporter. Bidirectional arrow means the MsmK and MalK ATPases can energize permeases interactively. Unidirectional arrows mean the degradation of pullulan and glycogen by pullulanases SpuA or ApuA.

It is well known that carbohydrate utilization is essential for the growth and pathogenesis of many pathogenic bacteria. As necessary nutrients, most carbohydrates must be transported into bacterial cells, and then used for different biological processes. There are mainly two carbohydrate transport systems in bacteria: the PTS system and ABC transporters. As an ABC transporter, two permease domains and two ATPase subunits are essential structural components. The two permeases form an integral membrane channel, and the two ATPase subunits energize the transport though ATP hydrolysis. Interestingly, some ABC transporters possess their own ATPase, while others share a common ATPase. For example, MsmK functions as a shared ATPase for three carbohydrate ABC transporters RafEFG, SatABC and MalXCD in *S*. *penumoniae* (Fig 7) [32,35]. *S*. *suis* is an important emerging zoonotic bacterium causing increasing dead infections in pigs and humans worldwide, especially in Southeast Asia and North Europe. Here, we identified MsmK as a shared ATPase energizing the transport of raffinose, melibiose, maltotetraose, glycogen and maltotriose in *S*. *suis*.

When screening the genome sequences of *S*. *suis* available in the GenBank, two genomic regions were found to encode the CUT1 subfamily of ABC transporters MsmEFG (SSUSC84_1397–1405) and MalXCD (SSUSC84_1932–1938) (Fig 1A and 1C), but no ATPase gene was found in and around these regions. The gene SSUSC84_1724, located between the MsmEFG and MalXCD in *S*. *suis* strain SC84 genome (Fig 2A), encoded a protein that displayed the highest similarity to the MsmK of *S*. *pneumoniae* (79.5%) and *S*. *mutans* (78.5%). It contains all the conserved domains of an ATPase (S1 Fig). ATPase assay also demonstrated that the SSUS84_1724 homolog in strain SC-19 indeed encodes an ATPase (Fig 2C). It could form a dimmer (Fig 2B), this agrees with its function in an ABC transporter (two ATPase subunits are needed) [27]. Given the high similarity of the transporters MsmEFG and MalXCD of *S*. *suis* to the carbohydrate ABC transporters MalXCD of *S*. *pneumoniae* and the MsmEFG and MalXFG of *S*. *mutans* (Fig 1), we annotated this ATPase (SSUS84_1724) as MsmK, and speculated that it functions as a shared ATPase for the MsmEFG and MalXCD transporters of *S*. *suis*.

To validate this hypothesis, an isogenic mutant and a genetic complementary strain were constructed. Fermentation and cultivation studies demonstrated that MsmK was essential in *S*. *suis* for the utilization of raffinose, melibiose, maltotetraose and glycogen, and involved in maltotriose utilization as well (Figs 3 and 4, Table 4). According to the structure similarity to the corresponding functionally-known ABC transporters of *S*. *pneumoniae* and *S*. *mutans*, qRT-PCR was performed to detect the expression of relevant genes (Table 3). The results showed that the MsmEFG transporter of *S*. *suis* was responsible for taking up raffinose and melibiose, while the MalXCD transporters was responsible for utilization of maltotriose, maltotetraose and other maltodextrins in *S*. *suis*. Carbohydrate uptake by MsmK and relevant transporters of *S*. *suis* was summarized in Fig 7.

Moreover, maltotriose was transported by the MalXEFG transporter and MalT, a phosphoenolpyruvate-dependent phosphotransferase in *S*. *mutans* [35,36]. In *S*. *pneumoniae*, the MalXCD transporter and MalT homolog (SP_0758) undertake this process [23]. while the MalT homolog (SSUSC84_0343) may be responsible for uptake of maltose and maltotrise in *S*. *suis*. Though the results of qRT-PCR, we found that MalT not only takes part in transport of maltriose, might also have a role in transport of maltotetraose in *S*. *suis* (Table 3).

Some polysaccharides can also be utilized by bacteria. For example, starch and glycogen can be degraded into glucose, maltose, maltotriose and other maltodextrins by *S*. *suis* bifunctional amylopullulanase ApuA [12], then the products may be transported by the MalXCD and MalT transporters. It has been reported that in the oropharyngeal cavity, the high amount of starch at mucosal surfaces support the growth of *S*. *suis* [11]. However, fermentation assays in this study indicate that MsmK mutant of *S*. *suis* still ferments starch but cann’t ferment glycogen any more (Table 4). This suggests that MsmK is essential for glycogen utilization, but not for starch utilization. During the experiment, we found that the color change of Δ*msmK* in starch occurred later that in other carbohydrates. The SpuA, the ApuA homolog exists in *S*. *pneumoniae*, can degrade glycogen into maltodextrins [23]. However, the SpuA/ApuA homolog is absent in *S*. *mutans*. *S*. *mutans* is an oral bacterium producing organic acids through fermentation of host-derived carbohydrates [53]. For saliva can degrade starch (the major dietary) into maltose and maltodextrins by α-amylase [54], *S*. *mutans* can ferment the products directly, thus it has no amylase homolog (Fig 8A).

*S*. *peumoniae* can take up exogenous sialic acid by the SatABC transporters, even cleave sialic acid as carbohydrate source during colonization [30,32]. Unlike *S*. *peumoniae*, *S*. *suis* cann’t grow on the CDM supplemented with sialic acid as sole carbohydrate (data not shown), although *S*. *suis* has been demonstrated to obtain sialic acid through *de novo* endogenous biosynthesis [55].

Carbohydrate utilization is essential not only for the growth, but also for pathogenesis of bacteria [30,32]. As a key component of the carbohydrate ABC transporters, MsmK may also play a role in the pathogenesis of *S*. *suis*. To this end, the ability of survival and colonization of Δ*msmK* and its parental strain SC-19 was tested in a mouse infection model. In the competition infection assays, Δ*msmK* showed a significantly reduced ability of survival and colonization in the brain of mice when compared with the parental strain (Fig 6). It is likely that the ability to utilize glycogen contributes colonization of *S*. *suis*. Several studies have established the importance of pathogen glycogen metabolism in environmental survival, symbiotic performance and colonization, even virulence [11,16–19,56]. The mammalian brain contains glycogen, which is located predominantly in astrocytes and the principal role for brain glycogen as an energy reserve [57,58]. Also, it has been described that in the organs and tissues, glycogen released from damaged cells can be degraded by ApuA and the breakdown products sustain growth of *S*. *suis* [11]. The Δ*msmK* strain couldn’t ferment glycogen or grown on glycogen as the sole carbohydrate, which may be one of the possible reasons for the defect colonization. It is worth noted that there was no significant difference on the bacterial load in mouse brain in the early stage (12 h) of infection by the Δ*msmK* and parental strains (Fig 6). This indicates that MsmK does not influence *S*. *suis* invasion.

In conclusion, MsmK is identified as an ATPase functioning as a key component of ABC transporters in *S*. *suis*. It is essential not only for the utilization of multiple carbohydrates including raffinose, melibiose, maltotetraose, glycogen and maltotriose, but also for the ability of *in vivo* survival and colonization of *S*. *suis*. Awareness of the important roles of carbohydrate utilization during the growth and infection of pathogenic bacteria may lead to new strategies to combat bacterial infectious diseases.

## Supporting Information

S1 Fig Alignment of protein sequences of MsmK of *S*. *mutans*, *S*. *suis* and *S*. *pneumoniae*. Gray boxes indicate the characteristic motifs of an ATPase.
(TIF)

S2 Fig Confirmation of the strains Δ*msmK* and CΔ*msmK*. Western blot results confirmed the MsmK inactivation in strain Δ*msmK* and complementation in strain CΔ*msmK*. The supernatant of bacterial lysis was separated by SDS-PAGE and probed with anti-MsmK serum or anti-Enolase serum in this assay.
(TIF)

S3 Fig Mutation of *msmK* has no effect on the growth of SC-19 in glucose. The SC-19 and Δ*msmK* strains were grown on CDM containing 1% glucose as sole carbon source. OD_600_ (A) and bacterial numbers (B) were measured every 2 hours. Data are means ± SD from at least three independent experiments.
(TIF)
