# Supplementary figures and images for: MsmK, an ATPase, Contributes to Utilization of Multiple Carbohydrates and Host Colonization of Streptococcus suis
Source: PLoS One. 2015 Jul 29;10(7):e0130792. doi: 10.1371/journal.pone.0130792 (PMC4519317; doi:10.1371/journal.pone.0130792)

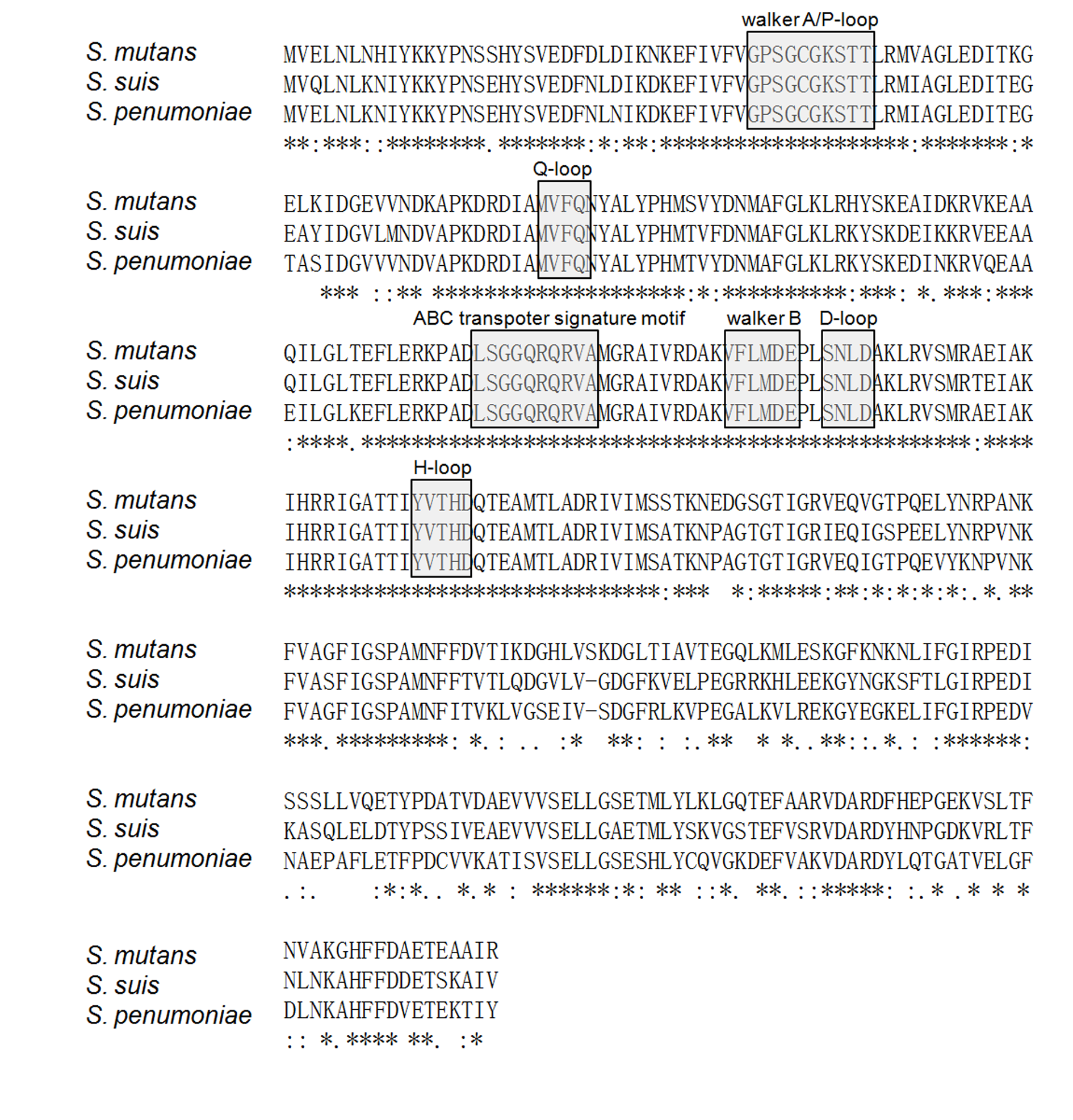

Supplement: S1 Fig — Gray boxes indicate the characteristic motifs of an ATPase. (TIF) [file pone.0130792.s001.tif]

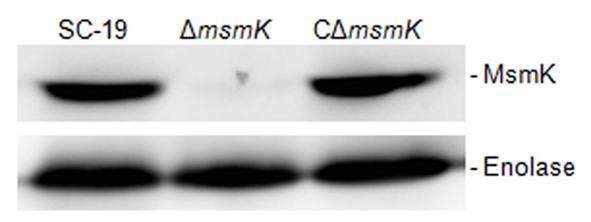

Supplement: S2 Fig — Western blot results confirmed the MsmK inactivation in strain ΔmsmK and complementation in strain CΔmsmK. The supernatant of bacterial lysis was separated by SDS-PAGE and probed with anti-MsmK serum or anti-Enolase serum in this assay. (TIF) [file pone.0130792.s002.tif]
